# Gender-specific responses to climate variability in a semi-arid ecosystem in northern Benin

**DOI:** 10.1007/s13280-016-0830-5

**Published:** 2016-11-22

**Authors:** Afiavi P. Dah-gbeto, Grace B. Villamor

**Affiliations:** 1 Federal University of Technology, PMB 65, Minna, Niger State Nigeria; 2 Center for Development Research (ZEF), Walter-Flex 3, 53113 Bonn, Germany

**Keywords:** Anticipatory learning, Coping strategy, Grazing game, Land-use preferences, Resilience

## Abstract

Highly erratic rainfall patterns in northern Benin complicate the ability of rural farmers to engage in subsistence agriculture. This research explores gender-specific responses to climate variability in the context of agrarian Benin through a household survey (*n* = 260) and an experimental gaming exercise among a subset of the survey respondents. Although men and women from the sample population are equally aware of climate variability and share similar coping strategies, their specific land-use strategies, preferences, and motivations are distinct. Over the long term, these differences would likely lead to dissimilar coping strategies and vulnerability to the effects of climate change. Examination of gender-specific land-use responses to climate change and anticipatory learning can enhance efforts to improve adaptability and resilience among rural subsistence farmers.

## Introduction

This study explores gender-specific responses to climate variability and related coping strategies in the context of agrarian Benin. There is only a limited understanding of gender-differentiated impacts of climate change in West Africa (Babugura et al. 2010), and therefore an urgent need to integrate gender analyses into climate change adaptation responses and more broadly in scientific research (Carr and Thompson 2014; Schiebinger 2014). Very few studies have explored linkages between gender and agro-ecological sustainability, decision making, and the development of multi-functional landscapes (Meinzen-Dick et al. 2014; Villamor et al. 2014, 2015). We explore the following three questions at a study site in northern Benin in order to contribute to the overall understanding of resilience among subsistence agricultural systems in semi-arid ecosystems: (1) How do male and female farmers perceive and react to climate variability and extreme weather conditions? (2) Do male and female perspectives differ in terms of land-use preferences and adaptation to climate variability? (3) What determines gender-specific decisions under conditions of climate uncertainty? Improved knowledge of gender-differentiated exposure and response to shocks contributes to anticipatory learning, which is the key in helping communities to become more resilient to risks and uncertainty associated with global climate change (Nuttall 2010; Kumar and Quisumbing 2014). This paper addresses the need to investigate the determinants of anticipatory learning in order to enhance resilience at the site level (Tschakert and Dietrich 2010).

### Why are gender-specific responses to climate change important?

Gender-specific responses to climate change impacts have largely been ignored in the context of international debates and policy frameworks (Denton 2002; Omari 2010). According to the 4th Assessment Report of the Intergovernmental Panel on Climate Change (IPCC 2007), most climate change impact studies in West Africa fail to consider gender disparities, particularly in respect to land use, management preferences, and related perspectives.

In Benin, 40 % of the GDP and 70 % of employment are associated with agriculture, and 70 % of females live in rural areas where they are responsible for 60–80 % of the agricultural work performed in the country and furnish up to 44 % of household subsistence labor (FAO 2012). The country is threatened by extreme climatic variability and weather conditions such as flooding, drought, and erratic rainfall patterns (Kpadonou et al. 2012). Although there are a few studies from Benin on climate perception and the vulnerability of rural farmers to the effects of climate change (Leal Filho et al. 2013; Yegbemey et al. 2013), gender issues related to climate change in the country have not been sufficiently addressed.

Emerging work on gender implications of climate change in agrarian settings highlights how gender-specific patterns of labor and responsibility result in differential vulnerability (Carr and Thompson 2014). Women in Benin are more vulnerable to the effects of climate change than men because of their locally defined responsibility for reproductive and domestic roles, limited access to natural resources, and role in decision making (Omari 2010; Djoudi and Brockhaus 2011). Consequently, men and women have different adaptive strategies and spatial perceptions that reflect their activities, social positions, and differential access to and control over resources (Kumar and Quisumbing 2014; Meinzen-Dick et al. 2014; Villamor et al. 2015). Examination of gender-specific differences improves understanding of the underlying issues, and can contribute to efforts to reduce gender inequity and related vulnerability.

### Experimental gaming and anticipatory learning

Anticipation is a critical component for building resilience (Boyd et al. 2015). By being proactive, anticipation focuses more on foresight drawn from predictive capabilities, knowledge, experience, and skills (Nuttall 2010). Botkin et al. (1979) distinguished anticipatory learning from adaptive learning, with adaptation being a reactive adjustment to change (or external pressure), and anticipation implying preparation for possible contingencies and consideration of long-term alternatives. In a non-anticipatory situation, people may simply wait for problems to worsen before seeking remedies, or may react and search for answers until it is too late to implement effective solutions (Botkin et al. 1979). In this sense, adaptive learning poses a limitation, and anticipation is required to adapt decisions and behaviors to a dynamic environment.

One approach that captures this perspective is the use of interactive board games. Although games have a long history of application in anthropology for educational and pedagogical purposes, both computer-based simulations and gaming are increasingly important tools for improving our understanding of adaptive behaviors to climate change and other scenarios (McAllister et al. 2006; Barreteau et al. 2007; Villamor et al. 2015). We explore gender-specific responses to climate variability and anticipatory capacity using a gaming experiment. In this scenario, farmers were segregated by gender and subjected to a highly stylized landscape in an experimental game that mimicked the erratic rainfall patterns and semi-arid conditions in Benin. This allowed farmers to dynamically conceptualize future possibilities stemming from climatic pattern instability in a way that can improve their adaptive and anticipatory capacities, increasing their appreciation of adaptive measures that are likely to be most feasible (Tschakert and Dietrich 2010).

We explore how decisions vary between genders and related dimensions of anticipatory learning. According to Rhea (2005), anticipatory learning has four defining dimensions: *foresight* (thinking ahead about how trends, issues, and developments observed in the present are likely to shape alternative futures), *identity* (how we act in the present, and what is important for us to create in the future), *direction setting* (forging the learning from foresight and identity into wiser decisions about what to create in the future and how to do it), and *innovation* (identifying strategies to avoid a feared future).

## Materials and methods

### Study area

**Fig. 1:**
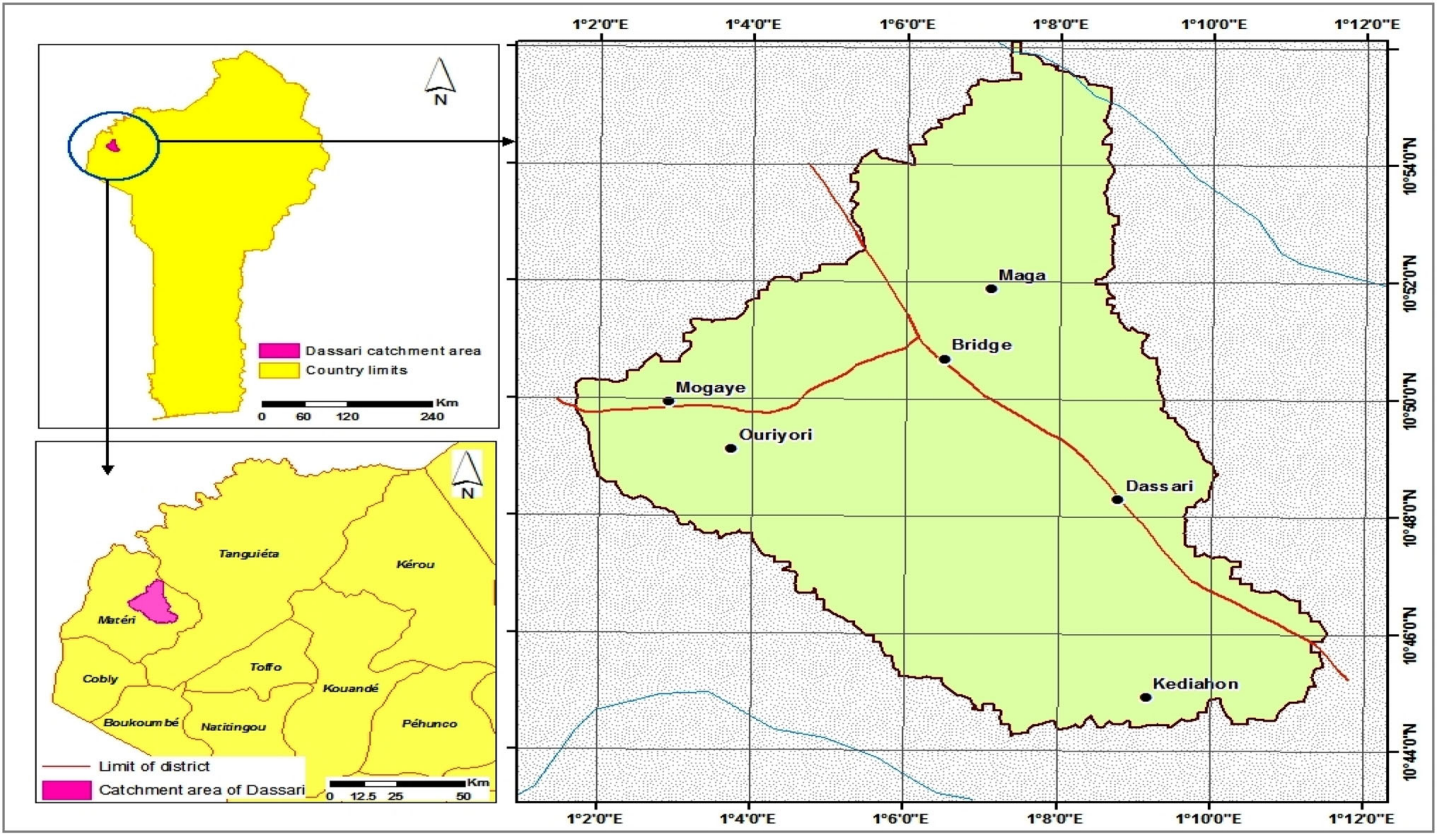
Location of the Dassari watershed in the Benin Republic

The study site is located in the Dassari watershed in northwestern Benin. The 589 km^2^ watershed covers most of the geopolitical district of Dassari located between 10°44′12″ and 10°55′48″ North latitude, and between 1°01′55″ and 1°14′54″ East longitude (Fig. 1). The maximum daily temperature varies between 34 and 40 °C, and the mean annual temperature is 27 °C. The population of Dassari was 24 891 in 2009 (Dah-gbeto 2014). The dominant ethnic group in the district is the Biali, and most livelihoods are agricultural. According to Sow et al. (2014), the Biali constitute over 90 % of the Dassari population and perceive themselves as the natives and rightful landowners in the area. Livestock is an important indicator of wealth (e.g., for bride price payments and funerals). Traditionally, the Biali are sedentary, but have been forced into migratory patterns in search of fertile land and seasonal economic activities as far as Nigeria (Sow et al. 2014). Most of the Biali believe in traditional ancestor worship, and adherence to cults and belief in mystical power is socially ubiquitous, which influences perceptions about the use of natural resources.

### Data collection

The household survey was conducted with a stratified random sample and designed with the objective of describing the respondents’ characteristics as well as related opportunities, constraints, and decisions related to climate change response. A total of 260 respondents (of which 197 were male and 63 were female households) were surveyed using a semi-structured questionnaire in February and March 2014. The participating women were land owners due to inheritance or household heads due to the absence of husbands engaged in (seasonal or permanent) migratory labor. The survey questionnaire covered respondents’ socio-economic characteristics, land-use preferences, and factors influencing choices regarding coping strategies and barriers to adaptation in the study area, as well as decisions about farm- and household-level adaptation strategies.

**Fig. 2:**
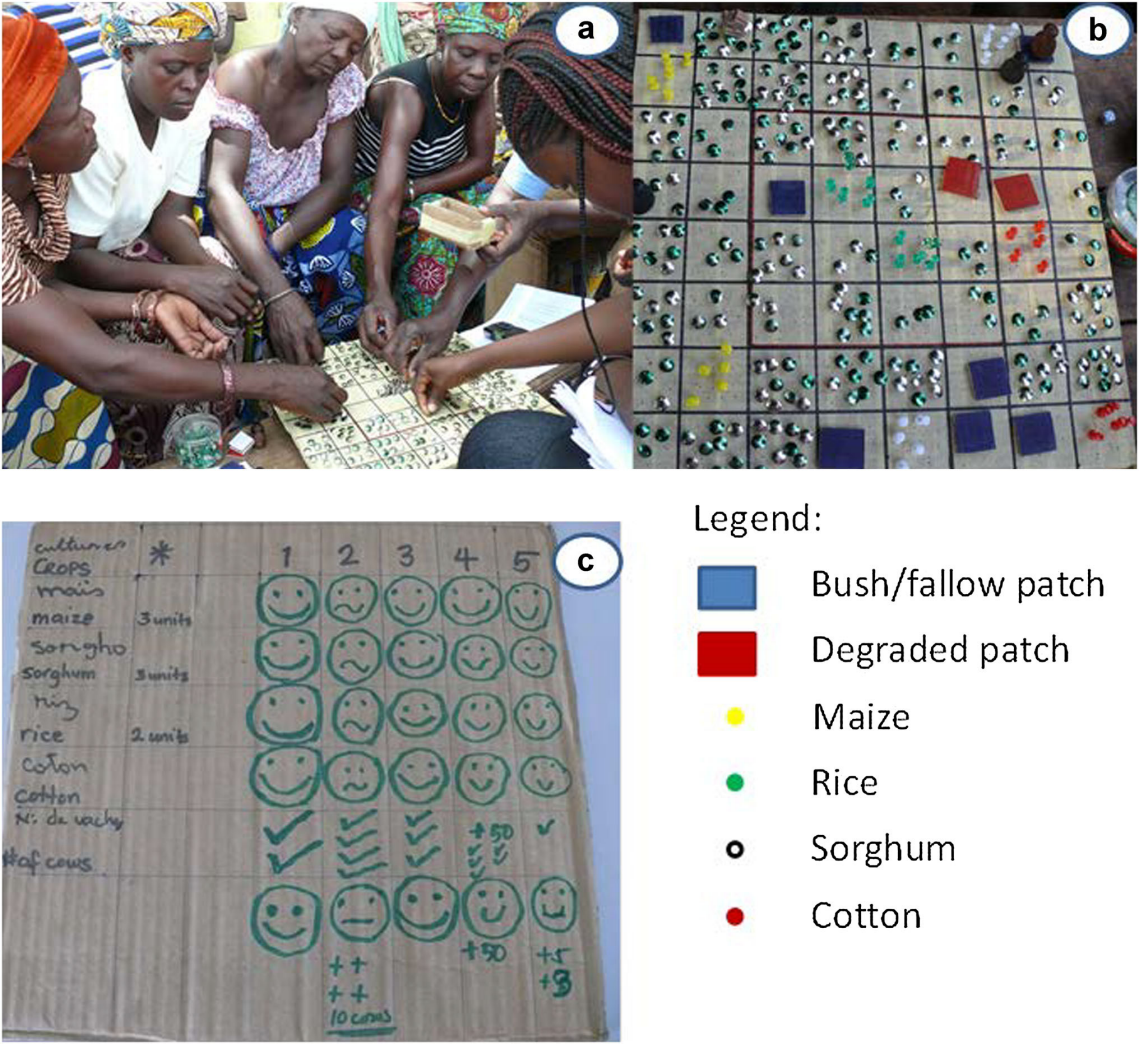
Sample images of women-only group (**a**), game board (**b**), and score sheet (**c**)

For the gaming experiment, we modified a board game called the “grazing game” developed by Villamor and Badmos (2016) that was field tested in a savannah area of Ghana. The purpose of the game exercise is to reveal the processes that lead to overgrazing and desertification, and to explore the adaptive strategies, local knowledge, and behaviors of participating farmers under drought conditions. The main modifications to the game were the explicit disaggregation of players by gender, potential for crop expansion as opposed to restricted expansion opportunities (as proposed by female players), and a simplified scoresheet (Fig. 2c).

A total of 6 games were played among 37 survey respondents who expressed willingness to participate. Players were subdivided into 3 men-only groups and 3 women-only groups corresponding to each of three villages. The main objective of this game exercise was to observe behaviors, perceptions, and responses of groups of male and female players (farmers) in response to erratic rainfall conditions in a dynamic group setting where players can openly and actively discuss options and respond to different scenarios. The game uses an 8 × 8 grid board (each cell or ‘patch’ measures 4 cm × 4 cm) that represents a farming landscape. Colored pins are used to represent livestock forage and crop units; 3 pebbles are used to indicate herds; the patches are color-coded to represent different land uses (e.g., degraded and bush or fallow land); and a score sheet is kept for each game (Fig. 2). Rainfall during each round is determined by a die, and players react accordingly. Each game consists of 5 rounds, each simulating an annual cycle. Players assume the role of farmers and attempt to manage herds to maximize production without causing land degradation, while also cultivating crops on some patches for subsistence purpose. Crop options include rice, maize, cotton, and sorghum (Fig. 2).

At the beginning of the game, each player has a herd of 5 livestock as their initial capital. Livestock refers generally to grazing animals found in the study area, which are locally considered wealth indicators, and may include donkeys, cattle, and goats. The detailed rules of the game (e.g., grazing, reproduction, regrowth of vegetation, and marketing) are described in Villamor and Badmos (2016). A degraded patch is created when grazing livestock consumes all of the forage or grass units, whereas a patch becomes ‘bush’ when the pasture units are left ungrazed with more than 12 pasture units. Two scenarios are examined during the gaming exercise: (1) population growth at year 3 (represented by the addition of a new household with a herd of 5 livestock), and (2) a fertilizer scenario (in which a conventional commercial fertilizer is offered to players in exchange for livestock at year 4 to permit recuperation of pasture and crops in degraded patches as an local government initiative).

At the end of every game, a reflection session is held to clarify and verify the strategies/decisions made by the players using multiple-choice and open-ended questions (Villamor and Badmos 2016). Each game is overseen by a game master who also facilitates the players introduction to the game and a reflection session following the end of each game. Two additional observers assist the group members in filling out the score sheets, take photographs of the game board at the end of each round, and record notes of the conversations among participants, including behaviors during key events (e.g., low rainfall, different scenarios, prior to casting the die to determine rainfall for each round). A digital recorder was also used to capture the results of each game.

### Data analysis

Household survey data were analyzed using the Statistical Package for Social Sciences (SPSS) version 14.0. The data collected from the grazing game exercises were processed and analyzed using an MS Excel spreadsheet. The indicators used to compare the outcomes of the game are as follows: (1) the number of degraded patches, (2) the total number of livestock produced, (3) the number of bush/fallow patches, (4) the number of cropland patches, (5) the number of livestock lost, and (6) income from livestock sales. To test outcome differences between genders for significance, we used a *t* test and a Wilcoxon rank-sum test using STATA 12.0.

The reflection exercise after each game is the most important part of the experimental game session in which learning is assessed (Thatcher 1990). During these sessions, the players are asked questions in order to re-evaluate their game experience and identify anticipatory learning dimensions or determinants. Questions include the following: What do we need to know about alternative futures (*foresight*)? What do we believe about the world and ourselves (*identity*)? What do we want to create in the future and how we will we do it (*direction setting*)? What solutions can we create together (*innovation*)?

## Results

### Descriptive characteristics

The respondents’ socio-economic characteristics are summarized in Table 1. The mean age of sampled individuals, regardless of gender, is approximately 52 years. On average, men have slightly higher income, more landholdings, and dependents relative to women (Table 1). The respondents have an average of almost 40 years of farming experience, and women typically have more farming experience than men. With regards to land-use decision making, 84 % of male respondents consider themselves the head of the household and landowner, whereas 15 % reported practicing joint decision making (Table 2). Of the 63 female respondents, 41 % indicated that their husbands are the principal household decision maker, 37 % indicated themselves as primary land-use decision makers, and 22 % practice joint decision making with their husbands. Women typically assume the role of household head upon the death of their husbands (in which case land is automatically inherited from the deceased spouse) as well as during periods when their husbands live away from home for off-farm jobs.

**Table 1:** Descriptive statistics of survey respondents by gender (2014)

Variable	Gender	Mean	Standard deviation	Min	Max
Age	Men	51	12.10	31	98
	Women	52	9.33	37	72
	Total	52	11.48	31	98
Farming experience (years)	Men	39	9.49	30	80
	Women	41	8.00	26	65
	Total	39	9.18	26	80
Household size (#)	Men	10	4.99	2	38
	Women	9	4.79	1	21
	Total	10	4.95	1	38
Total area of landholdings (ha)	Men	6	3.67	1	30
	Women	5	3.44	1	17
	Total	6	3.68	1	11
Number of dependents (#)	Men	6	3.96	1	27
	Women	5	2.77	1	12
	Total	6	3.82	1	27
Income (US$/year)*	Men	1924.80	1541.30	62.89	12 721.20
	Women	1837.48	1595.40	92.24	8570.23
	Total	1903.69	1551.90	62.89	96 481.20

Participants included 197 men and 63 women

* Based on an exchange rate of 1 US$ = 477 CFA (Franc)

**Table 2:** Demographic and land ownership characteristics, and decision making in the study area by gender (2014)

Key variable	Men (*n* = 197) # (%)	Women (*n* = 63) # (%)
Marital status		
Married	187 (95)	44 (70)
Divorced	4 (2)	3 (5)
Widowed	6 (3)	16 (25)
Education level		
Primary	43 (21)	3 (5)
Secondary	34 (17)	–
University	1 (1)	–
Adult class	1 (1)	–
None	118 (60)	60 (95)
Source of labor		
Family (only)	49 (25)	30 (48)
Family and hired	88 (44)	15 (24)
Family and community assistance	60 (31)	18 (28)
Land ownership		
Land rental	5 (3)	5 (6)
Purchased	–	–
Free installation	33 (16)	6 (10)
Inherited	159 (81)	53 (84)
Land-use decision makers*		
Male	167 (84)	26 (41)
Female	1 (1)	23 (37)
Joint	29 (15)	14 (22)

* Including adaptation strategies

The majority of women do not have any formal education (Table 2). Most agricultural labor is provided by family members; however, some external labor is required depending on activity levels, particularly when household males are working away from home and crops need to be cultivated or harvested. The majority of respondents inherited their land.

### Individual perceptions on climate change and adaptation measures

In general, men and women share similar perceptions of climate change, especially with respect to rainfall patterns (Table 3). Respondents of both genders report that winds have become more violent over time and destroy their crops, in addition to uprooting trees and damaging houses. There is also consensus between genders that the ‘Harmattan,’ cold—dry and dusty trade winds from the Sahara—and the dry season are no longer distinguishable.

**Table 3:** Gender-specific perspectives on climate variability based on the survey results

Indicator	Men (%) (*n* = 197)			Women (%) (*n* = 63)		
	Decrease	Increase	No change/do not know	Decrease	Increase	No change/do not know
Changes in temperature	12	86	2	5	92	3
Rainfall distribution	97	3	0	97	3	0
Impacts of wind speed	11	88	1	8	87	5

The most commonly reported climate change adaptation measures (Table 4) are the use of fertilizers and pesticides (54 % of both men and women), followed by variation of planting dates and repeated sowing (42 % of men and 57 % of women), the use of improved crop varieties (42 % of men and 56 % of women), and adopting different farming practices such as mechanization (40 % of men and 41 % of women). Male respondents are more likely to report the use of fertilizers and herbicides to cope with declining yields due to worsening weather conditions. Both men and women respondents express a preference for an improved variety of maize that reaches harvest in 75 days and a cowpea variety that can be harvested at 65 days. Furthermore, there is a consensus that Bambara groundnut (*Vigna subterranea*), a traditional food crop, is no longer suitable to the current climatic conditions.

**Table 4:** Adaptation measures used by farmers in the Dassari watershed of Benin by gender

Adaptation measures	Men (%) (*n* = 197)	Women (%) (*n* = 63)
Use of fertilizer and pesticide	106 (54)	34 (54)
Varying planting dates and repeated sowing	83 (42)	36 (57)
Adoption of new improved varieties	82 (42)	35 (56)
Improved farming practice	79 (40)	26 (41)
Use of green manure and compost	27 (14)	7 (11)
Mixed culture	8 (4)	13 (21)
Protection of yam seed	14 (7)	3 (5)
Others (e.g., expand land)	25 (13)	8 (13)

Numbers outside of parentheses represent the frequencies of responses and numbers within parentheses represent the corresponding percentages of respondents

### Gender-specific group response to rainfall variability

**Fig. 3:**
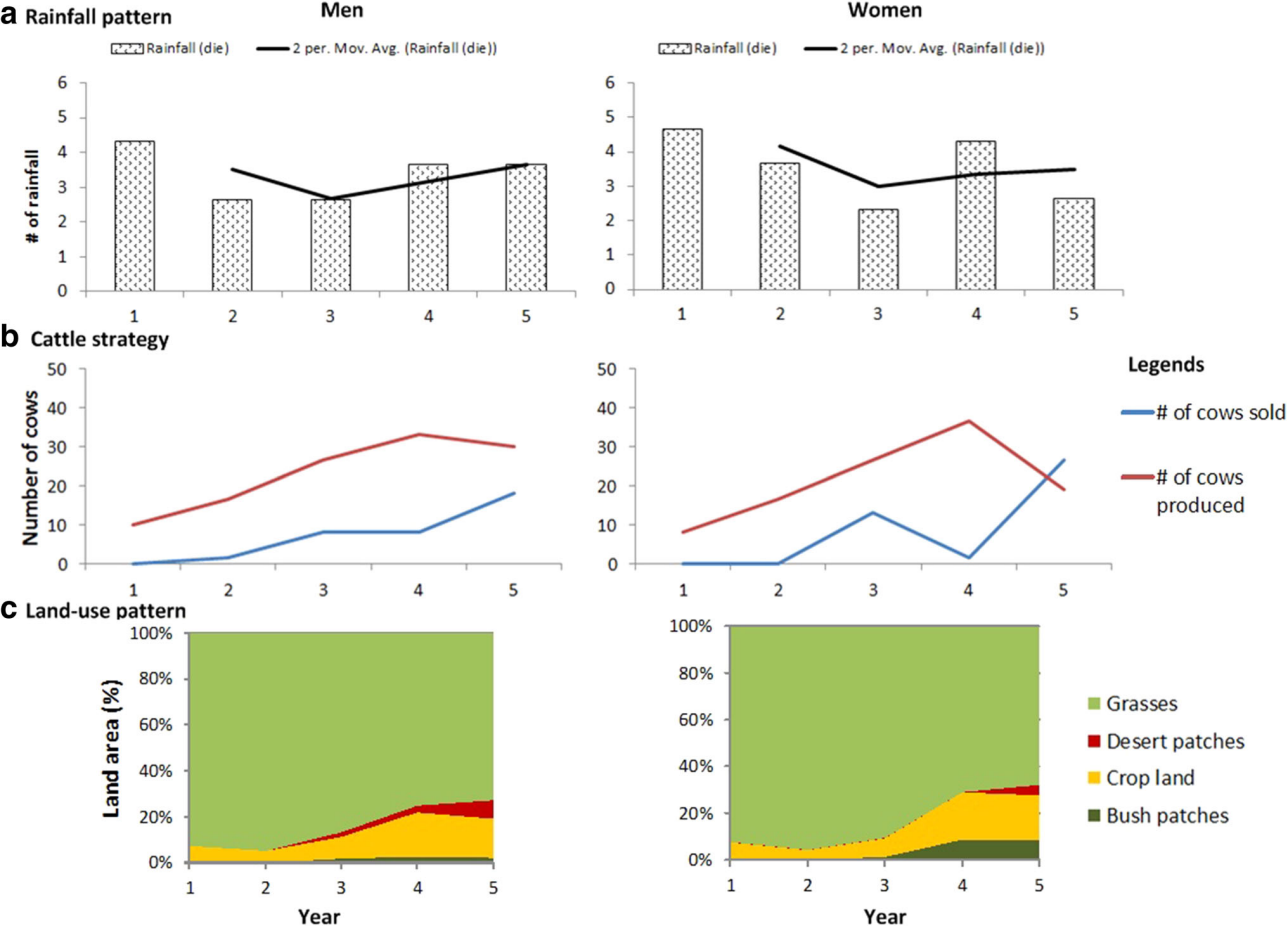
Game results with respect to **a** rainfall patterns, **b** grazing (cattle) strategy, and **c** land-use patterns by gender in the Benin study area (2014)

The gaming exercise results are summarized in Fig. 3. Neither the *t* test nor Wilcoxon test results indicate significant differences between genders. Groups of both genders have similar average rainfall patterns during the game exercises (Fig. 3a). During years 1–4 of each game, groups of both men and women have similar patterns of livestock production (Fig. 3b). The mode of selling livestock differs slightly between genders; women typically sell livestock in years 3 and 5, whereas men steadily sell livestock every year. Overall, women-only groups produce less than half of the average number of degraded patches as the men-only groups (Fig. 3c). Women-only groups convert more patches to crop production than men-only groups (Fig. 3c). Most women participants express displeasure when they produce bush/fallow or degraded patches (Fig. 3c), and make greater efforts to restore these patches by selling livestock and using the proceeds to purchase fertilizer or convert the patch to crop production. In contrast, men generally exhibit a more positive attitude regarding the production of bush patches, which they perceive would serve “somewhat like a fallow to restore soil fertility.” Although both men and women perceive cotton as superior to other crops (probably because it provides short-term income and is subsidized through discounted fertilizer and pesticide assistance by the government), women typically choose to cultivate staple crops such as maize and rice, which are not subsidized by the government.

Players respond to lower rainfall by dividing herds or by feeding their livestock half rations. Also, according to the farmers, “they always prepare their land before the rain starts, but nowadays the rain is delayed.” This statement from the post-game session implies that every decision depends on information regarding rainfall.

### Gender-specific perceptions and strategies

Both genders view low rainfall as ‘bad weather’ that leads to drought and starvation, and high rainfall is associated with abundant harvests and good conditions. Participants of both genders react competitively to incoming players under the population growth scenario by forcing newcomers to sell their livestock, especially when rainfall is low and livestock forage is less available. All groups engage in group consultations for making decisions, especially with respect to land-use and coping strategies. During the fertilizer scenario, only two (a male-only and a female-only) groups accepted the offer. Among those groups, the male-only group used the subsidized fertilizer to restore 10 degraded patches and the women-only group restored 2 degraded patches.

There are a number of apparent differences apparent from the gaming experiment (Table 5). Communication style among group members differed by gender. Men typically only have group discussions (about what coping strategies to adopt) during rounds with low rainfall, whereas participating women engaged in group discussions regardless of rainfall levels, suggesting that the women are generally more communicative than the men. Accordingly, women are more hesitant to make decisions individually and appear more likely to rely on others, such as neighbors. As a result, games among women-only groups lasted approximately 1 h longer than among men-only groups.

**Table 5:** Gender-specific reactions to game scenarios

Setting/scenario	Men’s reaction/response	Women’s reaction/response
Before throwing the die for rain	Players were quiet and waiting for the result of the die	Cite prayer for a good rain
When rainfall is low…	Player comments included “We are dead this year” and “the drought will be serious this year” Older households forced newcomers to feed their livestock half rations to reduce grazing pressure on pastures	Player comments included “You are going to die alone” in response to the player rolling the die, while another player stated “God have mercy on us, oh this year is going to be sad to us” Players spent more time in discussion, stressed the importance of divine intervention before initiating the game, and made greater effort to avoid degrading land Cash income from the sale of livestock was used to increase area for cotton and maize crops because they generate greater income for household expenditures (i.e., costs of sending children to school)
When rainfall is high…	The game rounds operate smoothly and quickly The players had little difficulty feeding livestock full rations and doubled the number of livestock at the end of each round	Players were very pleased and finished the round quickly
Population increase	Players were displeased and did not want to receive newcomers, one player’s reaction was “When the rain is misbehaving, we still need to feed more mouths…pity” Players sold more cattle and separated the remaining animals into 2 herds	Sell livestock to make space for crops and grazing
Crop choice	Yam, cotton, sorghum, and millet	Rice, maize, cotton, and vegetables (i.e., tomatoes, pepper, and okra)
Decision to sell/keep the livestock/crops	Mostly after throwing of die	Mostly after throwing of die
Game reflection	Inclusion of the protected area in the game, because “we could not expand our farms due to Pendjari Park”	Inclusion of pest and disease control component due to their effects on crop yields

The reflection sessions also revealed gender-specific spatial perceptions that affected their ability to adapt to climate variability. Men perceive that Pendjari National Park, a protected area that covers about 50 % of the study area, limits agricultural expansion and inhibits them from grazing their livestock (Table 5). Women are more likely to raise concerns about the incidence of crop pests and diseases near their homes that negatively affect yields and require additional control measures. They believe that pesticides would help them to protect their crops against pests observed under variable climatic conditions (Table 4). There are also gender-specific perspectives on crops. For example, yam is the most common crop choice among men and rice is most common among women.

Identified strategies for adapting to climate variability by both genders are improved herd management practices and farm improvements, while seeking governmental assistance is another common coping strategy. However, women generally consider livestock management as the responsibility of men. Women are more likely to sell small quantities of livestock during extreme weather events to improve livestock survival, while some express the desire to diversify income through building rental houses. Typically, women participants allocate income for household expenditures. Men are more likely to use proceeds from livestock sales to moving to larger towns or bordering countries (i.e., Nigeria) to seek wage labor opportunities, while some use the funds to purchase new motorcycles, improve their farms, and marry a new wife. The players make some recommendations related to policies and government assistance, such as increased subsidies. Most recommendations focus on water issues (mainly for drinking and agricultural activities), access to credit, fertilizer, drought resistant crop varieties, and extension services for improving farming practices, which supports the identified adaptation strategies generated from the survey results.

## Discussion

### Gender responses to climate variability: Insights on anticipatory learning

#### Foresight

Although the future is not predetermined, it can be shaped by countless events and conditions (as key forces) based on the choices made by individuals (Rhea 2005; Nuttall 2010). Based on the survey and experimental game results, men and women share similar perceptions on climate variability in the study area. They both perceive that weather patterns are changing, and that low rainfall threatens food security and can lead to starvation in the future. This observation reflects the conditions observed in the Sahel region of Africa during periods of drought and famine in the 1970s (1972–1974) and 1980s (1983–1985) (Le Barbé et al. 2002). There is also a consensus that temperature is increasing and that rainfall has become less frequent and predictable, which is supported by recorded trends in temperature and rainfall data from 1961 to 2010 (Dah-gbeto 2014). Droughts are perceived to be a result of the dynamics between low rainfall and inappropriate management decisions (e.g., overgrazing and expansion of cropland) among both men and women. However, the most consequential problem identified is increased rainfall variability, which affects crop calendars. As a consequence, farmers are being forced to abandon traditional crops or varieties in favor of shorter cycle alternatives.

However, participating men and women differed with respect to land-use preferences, which may affect their coping strategies for climatic variability over both the short and long terms. Women typically choose to cultivate staple food crops such as maize and rice (which are not government subsidized) to satisfy household subsistence needs, and that provide some surplus crops that can be easily sold at local markets. Currently, new maize varieties are being promoted as an adaptive strategy to climate change in West Africa, particularly shorter cycle varieties (Dah-gbeto 2014) that are often sold along with pesticides and chemical fertilizers. This could explain why women prefer the use of pesticides and fertilizers as a coping strategy for climate variability as shown in Table 4. Men typically select cotton as their favored crop. In the study area, the government offers credit for inputs related to cotton production. The agro-chemical inputs promoted or supported by government or private firms could shape the official image of the future in terms of subsistence farming practices among these men and women (Rhea 2005). According to Rubin (1998), this interpretation reveals the tone of the times and of the reality that people are living in. This image of the future may already be affecting important decisions about how they manage their farming systems.

Participating women-only groups perform better in avoiding patch degradation than men-only groups. Women groups sell livestock to prevent land degradation and use the proceeds for household improvements and to engage in other business activities. The women typically view livestock as a source of investment capital to expand cropland (Fig. 2b, c). In contrast, men-only groups are more concentrated on livestock production regardless of whether or not it results in land degradation. They sell livestock and often used the money to emigrate from the area in search of wage labor opportunities. These divergent attitudes suggest that women place greater value on their land as personal capital for producing food for subsistence purposes, while men place greater value on livestock production as a source of income that allows them to migrate. Currently, Benin is experiencing persistent population emigration to other West African countries due to difficult climatic conditions and dwindling natural resources (Sow et al. 2014). The data reflect this phenomenon, with more men, and particularly younger men, leaving households in search of off-farm employment. However, under these circumstances, women are more likely to be left at home to continue farming activities. For this reason, women are more vulnerable to climate change impacts, in part because their workloads increase dramatically due to emigration by household men. This may explain why women are more likely to cite prayer as a coping mechanism for shocks (Table 5).

#### Identity

This dimension examines how people orient themselves with regard to future possibilities, in ways that are bounded by values, beliefs, and emotions (Rhea 2005; Nuttall 2010). During the game, it was very apparent that women often follow spiritual traditions and religious rites. For example, at the end of every game round, members of women-only groups pray for improved rain during the next round, which was not observed in men-only groups. Although most Biali engage in traditional ancestor worship and expect ancestors to intercede on their behalf in the case of environmental issues (Sow et al. 2014), only women exhibit these behaviors during the gaming exercises. According to Rhea (2005), these religious and spiritual traditions offer learning practices for identity that can be refined by their experience. For these women, religious rites may be a way to avoid past negative experiences (especially when shocks are unbearable and they are powerless to cope, they seek solace in their faith in God). A similar study in southwestern Benin also found prayer to be a typical response to changing rainfall patterns (Baudoin et al. 2014).

Based on our current study design, we can also highlight other determinants such as awareness among both genders on climate change and their adaptation measures (Table 3) such as the use of fertilizers, and the adoption of improved crop varieties that could reflect a general willingness to engage with unknown yet conceivable risks. Access to information (i.e., weather forecasts) was reflected in the game by the result of each cast of the die. Members of both men and women groups wait for the die to be thrown before making decisions such as whether or not to sell livestock, plant crops, or purchase fertilizer (Table 5). According to Tschakert and Dietrich (2010), having access to learning spaces (in this case the game exercise) where alternative pathways can be tested and reflected upon makes reality and future uncertainty less daunting.

### Lessons learned and limitations of the study

The combination of a gender-disaggregated household survey with an experimental gaming exercise reveals the similarities and dissimilarities between men and women with respect to land-use-related strategies for adapting and coping with climate variability. The majority of the household survey results indicate similarities between men and women, especially on socio-economic aspects, whereas the experimental gaming exercise is useful for highlighting gender differences in terms of preferences, behavior (motivation), and decision making that shape their anticipatory capacity. In general, the gaming exercise provides a venue for women to share and negotiate changes in crop cultivation area and varieties, and to diversify their income sources (especially in this geographical context where women often withhold their perspectives when in the presence of men), while men are more likely to express their plans in the near future. The use of close-to-reality population growth and fertilizer subsidy scenarios is appropriate and realistic in the context of the study area, and is supported by advocates of adaptation studies because they help to examine not only climate change related risks, but also other overlapping pressures (Vermuelen 2014).

The gaming exercise requires considerable reflection and concentration, especially for the women because grazing livestock is normally the responsibility of men in the study area. In any event, this exercise enhances their anticipatory capacity with respect to grazing their livestock. In Mali, women are increasingly involved in livestock production because of persistent emigration of men from the population (Djoudi and Brockhaus 2011), which is also a possibility among Biali women. Nonetheless, participating women feel that playing the game helps them reconsider how to manage their livestock herds when facing situations like drought.

Of the four defining dimensions of anticipatory learning, only evidence of forecast and identity was found. One of the reasons for this outcome (which can also be seen as a limitation of this study) is that the two other dimensions require considerable time and multiple iterations (to grapple with incomplete information and uncertainties), and would require modification of the game design in order to target other future pathways. Furthermore, there is a need to couple the game exercise with additional tools such as participatory scenario building and future envisioning exercises to help identify innovative solutions (Rhea 2005; Badmos et al. 2014; Tschakert et al. 2014). However, because some of the determinants of anticipatory learning are challenging to measure, a follow-up study is needed that targets these specific dimensions. Nevertheless, it is important to reflect on the anticipatory capacity of local people, as most adaptation studies draw attention to technologies, regulation, policies, and practices that enable society to adapt to change (O’Brien 2012). In this study, an important social variable that affects resilience with a slow rate of change—*identity*—is found to be strong among women, and this is often overlooked and yet essential to understanding their resilience (Folke et al. 2010).

## Conclusions

This study explores gender-specific responses with respect to land use and coping strategies under conditions of increasing rainfall variability in the Dassari watershed of Benin using a household survey and an experimental gaming exercise. The methods are used to explore anticipatory learning that could foster societal resilience to the effects of climate change. In the gaming exercise, both male and female farmers played the role of land manager under erratic rainfall conditions. Both methods capture some aspects of the realities they are facing and common problems in the study area. Although perceptions and adaptation measures related to climate change are quite similar between men and women in the study area, the means, capabilities, and motivations vary by gender. Thus, their approaches to risk and uncertainty are also different. While men remain the primary decision makers in Benin households when they are around, women were found to respond in more active, dynamic, and innovative ways (in terms of diversifying income sources) when dealing with rainfall variability. Men continue to engage in seasonal migration or permanent relocation for work purposes, which is a common response to economic hardship. Although resulting migration or relocation may increase household resilience for both those who remain and those who emigrate, women are most likely to remain and will continue cultivating crops for household subsistence, thus bearing much of the impacts of climate change. Identity, as one of the defining dimensions of anticipatory capacity, was found to be strong among women in the study site, and is essential for understanding their resilience to climate change.
